# Transcriptome profiling of transgenic potato plants provides insights into variability caused by plant transformation

**DOI:** 10.1371/journal.pone.0206055

**Published:** 2018-11-08

**Authors:** Dae Kwan Ko, Satya Swathi Nadakuduti, David S. Douches, C. Robin Buell

**Affiliations:** 1 Department of Plant Biology, Michigan State University, East Lansing, Michigan, United States of America; 2 Department of Plant, Soil, and Microbial Sciences, Michigan State University, East Lansing, Michigan, United States of America; 3 MSU AgBioResearch, East Lansing, Michigan, United States of America; 4 Plant Resilience Institute, Michigan State University, East Lansing, Michigan, United States of America; Institute of Genetics and Developmental Biology Chinese Academy of Sciences, CHINA

## Abstract

Crop genetic engineering involves transformation in which transgenic plants are regenerated through tissue culture manipulations that can elicit somaclonal variation due to mutations, translocations, and/or epigenetic alterations. Here, we report on alterations in the transcriptome in a panel of transgenic potato plants engineered to be herbicide resistant. Using an inbred diploid potato clone (DMRH S5 28–5), ten single-insert transgenic lines derived from independent *Agrobacterium*-mediated transformation events were selected for herbicide resistance using an allelic variant of acetolactate synthase (*mALS1*). Expression abundances of the single-copy *mALS1* transgene varied in individual transgenic lines was correlated with the level of phenotypic herbicide resistance, suggesting the importance of transgene expression in transgenic performance. Using RNA-sequencing, differentially expressed genes were identified with the proportion of genes up-regulated significantly higher than down-regulated genes in the panel, suggesting a differential impact of the plant transformation on gene expression activation compared to repression. Not only were transcription factors among the differentially expressed genes but specific transcription factor binding sites were also enriched in promoter regions of differentially expressed genes in transgenic lines, linking transcriptomic variation with specific transcription factor activity. Collectively, these results provide an improved understanding of transcriptomic variability caused by plant transformation.

## Introduction

Advances in genetic engineering, generally defined as a method to directly modify the genome of an organism using biotechnology [1], have the promise to overcome limitations of conventional breeding in many important crop species [2–4] including clonally propagated crops, such as potato, fruit trees, and ornamentals, as it can bypass the challenges of inbreeding depression, long breeding cycles, and sexual incompatibility. Cultivated potato (*Solanum tuberosum* L.), the fourth most important food crop in the world following rice, maize, and wheat [5], is a unique system for genetic engineering due to its amenability to plant transformation and tissue culture [4, 6]. Indeed, the Colorado potato beetle-resistant potato, NewLeaf^TM^, commercialized by Monsanto in 1995 [6] was one of the first genetically engineered commercial crops. Two decades later, the first generation of Innate potatoes, engineered to reduce bruising and browning by RNA interference was approved by United States Department of Agriculture (USDA) Animal and Plant Health Inspection Service (APHIS) and released in 2014 by the J.R. Simplot corporation [7].

One widely used method to create genetically engineered crops is *Agrobacterium*-mediated transformation in which transfer-DNA (T-DNA) from *Agrobacterium tumefaciens* is inserted into the plant nuclear genome [8, 9]. Despite its versatility, *Agrobacterium*-mediated transformation is associated with limitations including genotype-dependent efficiency, non-T-DNA vector backbone transfer, non-targeted insertion of T-DNA into the host genome, and deletion of plant DNA at the insertion site [10–12]. One unintended effect of T-DNA insertion is termed the ‘position effect’ that impacts transgene expression [13] due to the influence of local epigenetic states and/or adjacent plant DNA sequences as demonstrated with the transgene, β-glucuronidase (GUS), in which activity varied from 0 to 6.7 nmol/mg/min among independent tobacco transgenic lines [14]. T-DNA insertion within a coding region, *cis*-regulatory element or an enhancer can lead to mutations. Furthermore, the 35S promoter near the left border (LB) or right border (RB) within the T-DNA could increase expression of a downstream gene as shown in activation tagging experiments of *Arabidopsis thaliana* in which enhancers in the T-DNA affect expression of neighboring genes [15]. While these impacts are due to transgene insertion, the process of tissue culture and regeneration of intact plants can also lead to mutations [16, 17] including changes in the epigenome that are associated with altered gene expression levels [18]. Indeed, there have been reports of unexpected physiological and biochemical phenotypes in soybean transgenic lines [19–21], including abnormal stem growth and yield reduction [22]. As a consequence of phenotypic variation associated with transformation, it is common practice to screen multiple transgenic lines to obtain the most robust line for commercialization.

Here, we investigate the impacts of plant transformation on the transcriptome in clonally propagated diploid potato engineered for herbicide resistance. Ten single copy T-DNA transgenic lines, originating from independent transformation events using a mutated acetolactate synthase 1 (*mALS1*) gene that confers herbicide resistance, were examined for the impact of transformation on the transcriptome and how this may be linked to phenotypic variation in transgenic lines.

## Materials and methods

### Plant material and growth conditions

A self-compatible S5 inbred line (DMRH S5 28–5) which was derived from a hybridization between a doubled monoploid, *S*. *tuberosum* Group Phureja DM1-3 516 R44, and a heterozygous diploid breeding line, *S*. *tuberosum* Group Tuberosum RH89-039-16, was used in this study for transformation [23]. Tissue culture plants were propagated from shoot tip and axillary bud explants and grown in Murashige and Skoog (MS) medium (PhytoTechnology Laboratories, Shawnee Mission, KS) and 3% sucrose (Sigma-Aldrich, St. Louis, MO) in Magenta G3 boxes (Magenta Corp., Chicago, IL) on fluorescent light racks set to 16 h light:8 h dark with a temperature of 25°C (light) and 18°C (dark) and a light intensity of 300 μmol m^−2^ s^−1^.

### Plasmid construction

**Fig 1:**
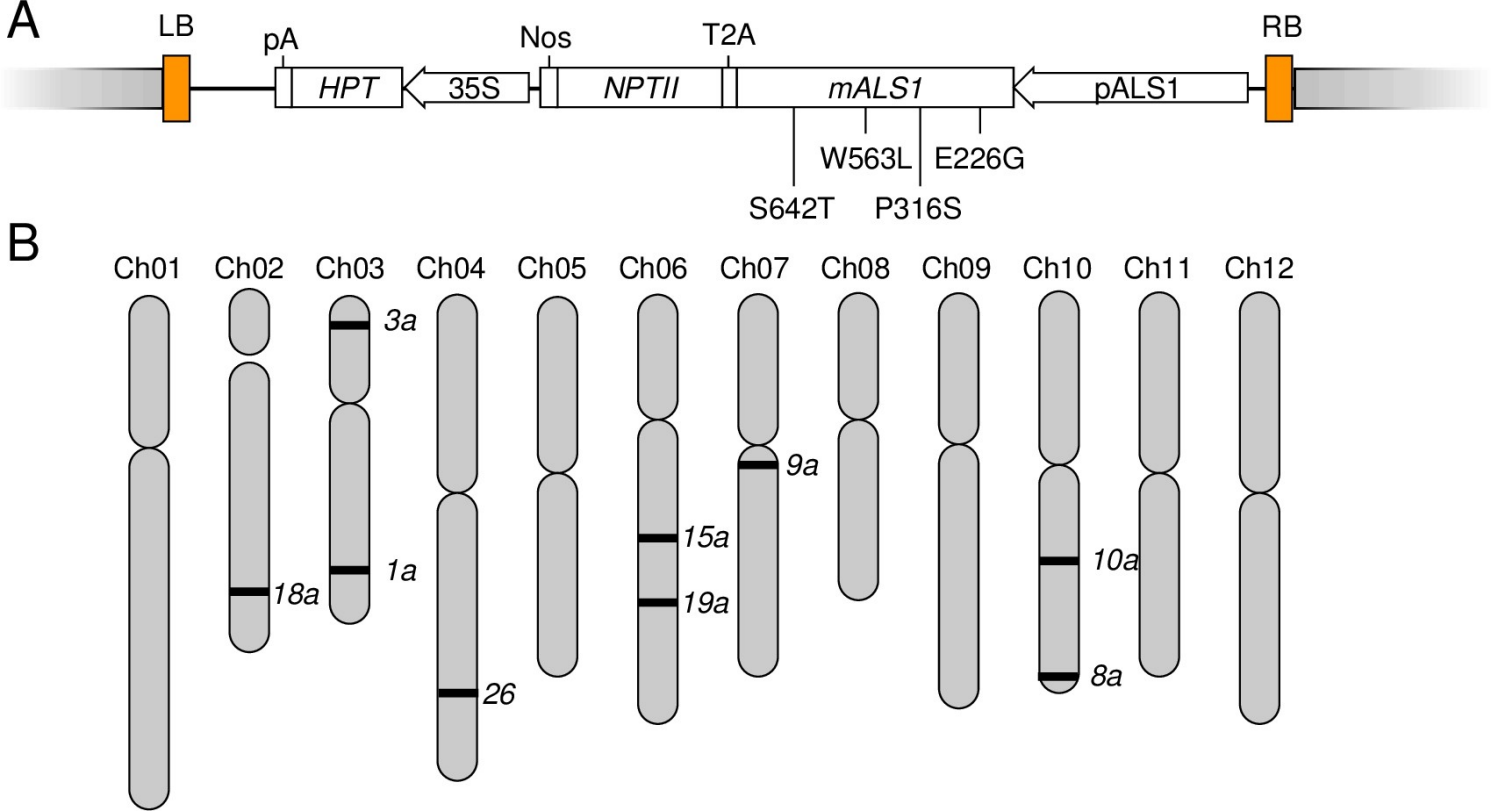
Creation of *mALS1* T-DNA transgenic lines. (A) A schematic representation of the T-DNA construct inserted into the plant genome. The T-DNA, in which *mALS1* is fused with *NPTII*, is driven by a native *ALS1* promoter. Grey boxes flanking the LB and RB indicate plant DNA. HPT, hygromycin phosphotransferase; NPTII, neomycin phosphotransferase II, Nos, terminator of nopaline synthase; pA, poly-A site; T2A, 2A peptide from *Thosea asigna* virus capsid protein. Four nonsynonymous mutations in *mALS1* are shown. (B) Location of T-DNA insertion sites in the DMRH S5 28–5 genome identified by inverse PCR approach. Size of each chromosome is proportional to its physical size. Black lines indicate locations of T-DNA insertion in transgenic lines. Note that the true location of the T-DNA insertion site in *als1-17* is unknown due to multiple mapping of reads derived from the inverse-PCR fragment to the genome. Full information of T-DNA insertion sites is provided in S1 Table.

The plasmid used in transformation was described previously [24]. In brief, a functional *ALS1* coding sequence incorporating W563L and S642T mutations driven by 2.5 kb native promoter region upstream of the endogenous *ALS1* along with T2A:*NPTII* translational fusion were cloned into the Gateway-compatible binary vector, pMDC32 (ABRC# CD3-738). A schematic representation of the T-DNA structure is shown in Fig 1A. The binary vector containing the mutated *ALS1* (*mALS1*) was introduced in *A*. *tumefaciens* GV3101 (pMP90) strain by electroporation using a Bio-Rad Gene Pulser (Bio-Rad, Richmond, CA) according to the manufacturer’s instructions.

### Plant transformation and regeneration

*Agrobacterium*-mediated transformation of potato explants was performed as previously described with some modifications [25]. Stem and leaf explants excised from 3-4-week-old healthy *in vitro* plants of DMRH S5 28–5 were chosen for materials due to their ease in transformation compared to other tissues such as microtubers, and placed horizontally on callus induction medium (MS with 3% sucrose supplemented with 0.9 mg/L thiamine-HCl, 0.8 mg/L Zeatin riboside ZR and 2 mg/L 2,4-D) for two days. An *A*. *tumefaciens* culture with an OD_600_ of 0.7 was used for inoculation of explants along with 50 μM acetosyringone. After 48 h of co-cultivation, the ex-plants were washed with liquid MS with 2% sucrose media containing 250 mg/L cefotaxime (Cef) and 150 mg/L Timentin (Tim). After blotting dry, explants were placed on shoot induction media, MS + 3% sucrose supplemented with 0.9 mg/L Thiamine-HCl, 0.8 mg/L ZR, 2 mg/L GA3, 150 mg/L Tim, 250 mg/L Cef and 50 mg/L kanamycin (Kan). Shoots (1–2 cm) regenerated from the calli were excised and put in tubes with MS + 3% sucrose media containing Cef, Tim and Kan. All regeneration events that rooted on selection media were taken for further molecular characterization.

### PCR screening and southern blot analysis

Genomic DNA was extracted from the leaves of regenerated events using the DNeasy Plant Mini Kit (Qiagen, Valencia, CA). Samples were PCR-screened using ALS1_homF1_m and NPTII start-R primers (S2 Table). Selected PCR positive events were further screened for single-copy insertion by Southern blotting using *NPTII* as a probe. The probe was synthesized and labeled using PCR DIG Probe Synthesis Kit (Roche Applied Science, Indianapolis, IN); the plasmid DNA vector (100 pg) used for plant transformation was amplified using NPTII Start-F and NPTII-R primers (S2 Table) according to manufacturer’s instructions. Genomic DNA for Southern blotting was isolated from 2 g of fresh leaf tissue of the selected *mALS1* T-DNA lines using a CTAB (cetyltrimethylammonium bromide) method [26]. Approximately 20 μg of genomic DNA from each sample was used for Southern blot analysis. Southern blotting was performed as previously described [25]. Genomic DNA was digested with *Hind*III and separated by electrophoresis on a 1% agarose gel, denatured, neutralized, and blotted onto a positively charged Hybond N+ membrane (GE Healthcare, Piscataway, NJ). Nucleic acids were fixed to the membrane by auto-crosslinking (Stratagene, La Jolla, CA) and chemiluminescent detection was performed using the CDP-Star system (Roche Applied Science, Indianapolis, IN). Plants containing a single insertion of the T-DNA were clonally propagated on root induction medium [MS + 3% sucrose, 1X Nitsch & Nitsch vitamins supplemented with Kan (50 mg/L; Sigma-Aldrich, St. Louis, MO), Cef (250 mg/L) and Tim (150 mg/L)].

### Identification of T-DNA insertion sites

Terminal leaflets of two-week-old plants grown in soil at 300 μmol m^−2^ s^−1^ under 16 h light:8 h dark with temperature 25°C (light) and 18°C (dark) for two weeks were used for genomic DNA. Two μg of genomic DNA isolated from individual transgenic lines and the wild-type (WT; DMRH S5 28–5), was digested with *Eco*RI that cuts just inside the right border or with *Rsa*I that cuts within the *mALS1* coding region. Digested DNA was purified using 1.8 X volume of AMPure XP beads according to manufacturer’s instruction (Beckman Coulter, Brea, CA), self-ligated using Quick Ligation Kit (New England BioLabs, Beverly, MA), and then purified as described above. Two μl of the resulting DNA was used for the 1^st^ round of PCR in which products were diluted by 5-fold and subjected to a 2^nd^ round of PCR. PCR reactions were performed using Phusion High-Fidelity DNA Polymerase (New England BioLabs, Beverly, MA) using these cycle conditions: 30 sec at 98°C, 20 cycles of 10 sec at 98°C and 3 min at 72°C, and 5 min at 72°C. A list of T-DNA-specific primer pairs is provided in S2 Table. The products of 2^nd^ round PCR were Sanger-sequenced which were then aligned to the potato reference genome v4.04 pseudomolecules using BLASTN in Spud DB [27].

### Herbicide spray assay

Two-week-old plants from tissue culture were transferred to soil and grown under 16 h light:8 h dark with temperature 25°C (light) and 18°C (dark) for 10 days in a growth chamber. Each line was replicated three times with each pot representing one replication. The plants were grown in a randomized design and rotated on a daily basis to avoid growth chamber positional effects. An aqueous solution containing 1 X of RAPTOR WG HERBICIDE (BASF, Triangle Research Park, NC), 1% (v/v) crop oil concentration (Loveland Products, Loveland, CO), and 2.5% ammonium sulfate (Winfield Solutions, Oakley, MI) was applied at 187 liters per hectare (L/ha), which is equal to 20 gallons per acre, using a moving-spray nozzle. For a positive control, we used the herbicide-tolerant transgenic line, *R32*, that was previously generated using the same *mALS1* binary vector in the potato line MSX914-10 and confirmed to have a strong herbicide resistance through three copies of the transgene on the genome (S1 Fig). Ten days after the herbicide application, all visible leaves of three replicate plants were scanned. Damaged areas were quantified using the ImageJ software (https://imagej.nih.gov); the experiment was independently replicated with consistent results.

### RNA-sequencing and analysis

Total RNA was extracted from terminal leaflets of two-week-old plants grown in soil at 300 μmol m^−2^ s^−1^ under 16 h light:8 h dark with temperature 25°C (light) and 18°C (dark) using the Qiagen RNeasy Plant Mini Kit (Qiagen, Valencia, CA) according to the manufacturer’s instruction. RNA-sequencing (RNA-seq) libraries were constructed using the Kapa Stranded RNA-seq Library Preparation Kit (Kapa Biosystems, Wilmington, MA) and sequenced in single-end mode on the Illumina HiSeq 4000 platform (50-nt) at Research Technology Support Facility Genomics Core at Michigan State University. Read quality was evaluated using FastQC (version 0.11.5) (http://www.bioinformatics.bbsrc.ac.uk/projects/fastqc). Reads were cleaned for quality and adapters with Cutadapt (version 1.8.1) [28] using a minimum base quality of 20 retaining reads with a minimum length of 30 nucleotides after trimming. Quality-filtered reads were aligned to the potato reference genome v4.04 [29] using Bowtie (version 2.2.4) [30] and TopHat (version 2.0.14) [31] with a 10 bp minimum intron length and 15,000 bp maximum intron length. Fragments per kilobase exon model per million mapped reads (FPKM) were calculated using the PGSC gene model annotation with Cufflinks (version 1.3.0) [32]. Transcript read counts were measured at the gene level using HTSeq (version 0.6.1p1) [33] in the union mode with a minimum mapping quality of 20 with non-strand-specific counting. Differential gene expression analysis was performed in each transgenic line relative to WT using DESeq2 (version 1.16.1) [34] within R (version 3.4.0). Genes for which expression differed with an adjusted *P*-value less than 0.05 were considered differentially expressed genes. Allele specific expression of *ALS1* and *mALS1* was measured at the four nonsynonymous mutations (Fig 1A) in all transgenic lines and WT by searching quality-filtered reads using the Vmatch alignment tool (http://www.vmatch.de).

Gene ontology (GO) enrichment analysis was performed using agriGO (version 2.0) (http://systemsbiology.cau.edu.cn/agriGOv2/) [35] with a false-discovery rate adjusted *P*-value < 0.05 (hypergeometric test) as a cutoff. Biological process GO categories were analyzed and the heatmap of GO analysis was produced using R package ggplots. *De novo* motif analysis was performed with 1,000 bp upstream sequence of the predicted transcription start site (TSS) using MEME (http://meme-suite.org/tools/meme) [36]. Enriched motifs identified in the MEME were filtered at E-value cutoff of 1E-07 and with occurrence at ≥ 10 genes. Similarity of enriched motifs with the *A*. *thaliana* DAP motif database [37] was assessed using TOMTOM [38].

### Data availability

RNA-sequencing datasets are available in the National Center for Biotechnology Information under BioProject PRJNA434568.

## Results and discussion

### Creation of potato plants genetically engineered for herbicide resistance using *Agrobacterium*-mediated transformation

To create herbicide-resistant transgenic potato plants, the *ALS1* gene which plays a key role in the branched-chain essential amino acids biosynthesis pathway [39, 40] and is a target trait that could be easily tested, replicated and quantified, was mutated to confer herbicide resistance (*mALS1;* Fig 1A). Using *Agrobacterium*-mediated transformation, the *mALS1* gene was introduced in the inbred diploid hybrid clone, DMRH S5 28–5, derived from a cross between the doubled monoploid DM 1–3 R44 which served as the reference genotype for the potato genome sequence [41], and RH89-039-16, a heterozygous diploid breeding line [41, 42]. The *mALS1* transgene contains two missense mutations, known to confer high levels of tolerance to imidazolinone herbicides in various crops [43] along with two other missense mutations, and is driven by a native promoter (Fig 1A). Following *Agrobacterium*-mediated transformation, shoots were selected, regenerated, and Southern blotting performed (S1 Fig); 10 transgenic lines, each with a single copy of the T-DNA in an independent transformation event, were used in downstream studies.

### Characterization of T-DNA insertion sites

To identify T-DNA insertion sites in the plant genome, we adopted an inverse PCR approach [44] and sequences adjacent to the RB of the T-DNA were amplified and sequenced. With the exception of *als1-17* [41], sequences flanking the T-DNA insertion sites in individual transgenic lines uniquely mapped to the potato reference genome confirming that our transgenic lines had independent T-DNA insertion events (Figs 1B and S2 and S2 Table). For *als1-17*, flanking sequences aligned to multiple locations, and as a consequence, we are unable to define its true location. T-DNA insertion sites were random across the 12 potato chromosomes, consistent with previous reports of random T-DNA integration [45]. In all cases, the T-DNA was inserted in an intergenic region (S1 Table). The distance between the T-DNA insertion site and the closest gene ranged from 200 bp to 237,643 bp, potentially contributing to variation associated with T-DNA positional insertion effects. Insertions of the T-DNA in *als1-3a* (Chr03: 7,593,471), *als1-10a* (Chr10: 44,433,457) and *als1-19a* (Chr06: 55,364,217) were < 5 kb away from the closest gene whereas the T-DNA insertion in *als1-1a* (Chr03: 42,911,657), *als1-18a* (Chr02: 46,573,347) and *als1-26* (Chr04: 64,830,170) were located > 5 kb but < 10 kb from the closest gene. Finally, insertions of the T-DNA in *als1-9a* (Chr07: 20,075,075) and *als1-15a* (Chr06: 39,382,926) were located > 50 kb away from the closest gene. The T-DNA insertions in these lines may affect expression of the closest gene via potential disruption of enhancer elements, which, in general, are distantly located from transcription start sites.

### Phenotypic variation in *mALS1* T-DNA transgenic lines

**Fig 2:**
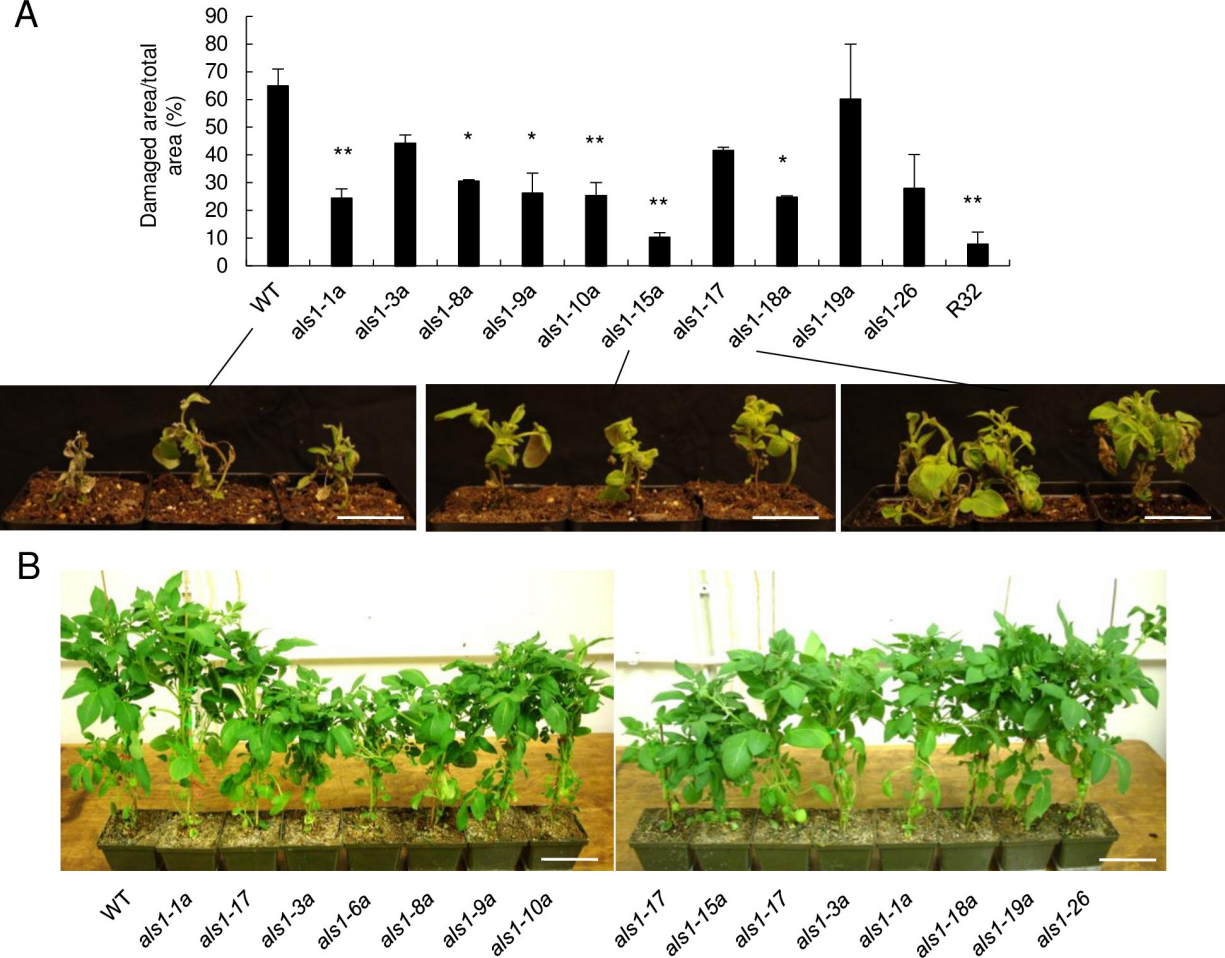
Phenotypic variation in *mALS1* T-DNA transgenic lines. (A) Histogram of herbicide tolerance of *mALS1* T-DNA transgenic lines. Two-week-old plants were sprayed with an imidizolinone herbicide in a spray chamber (mean ± SEM, n = 3). All leaves were scanned 10 days after treatment. Representative pictures of plants are shown below the histogram. Significant differences between WT and transgenic lines were calculated using a two-tailed Student’s *t*-test, *P-value < 0.05 and **P-value < 0.01. Scale bar = 50 mm. (B) Quantitative variation on plant height in *mALS1* T-DNA transgenic lines. The photo of plants was taken prior to flowering with no herbicide treatment. Scale bar = 100 mm. Note that *als1-6a* was removed from downstream analyses as the exact location of T-DNA could not be confirmed due to repetitive sequences in the inverse PCR products.

Variation in herbicide resistance conferred by the *mALS1* gene in transgenic lines was determined by quantitating the leaf area damaged 10 days after treatment with imidizolinone herbicide. Sensitivity varied from 10% to 65% of the leaf area damaged in transgenic DMRH S5 28–5 lines, while WT showed the greatest herbicide damage and R32, an herbicide-tolerant transgenic line used as a positive control (see Materials and methods), showed the least damage (Fig 2A). Among the 10 transgenic lines, six were significantly tolerant to the herbicide treatment (*als1-1a*, *P* = 0.009; *als1-8a*, *P* = 0.029; *als1-9a*, *P* = 0.015; *als1-10a*, *P* = 0.008; *als1-15a*, *P* = 0.008; *als1-18*, *P* = 0.006; *als1-18a*, *P* = 0.022). Three lines displayed a moderate tolerance [lower sensitivity than WT, but not statistically significant) (*als1-3a*, *P* = 0.058; *als1-17*, *P* = 0.056; *als1-26*, *P* = 0.076)]. One line displayed limited tolerance to the herbicide (*als1-19a*) comparable to WT. Interestingly, we noticed that growth rate, represented by plant height, also varied among transgenic lines (Fig 2B).

### Transcriptome profiling of *mALS1* T-DNA transgenic lines

**Fig 3:**
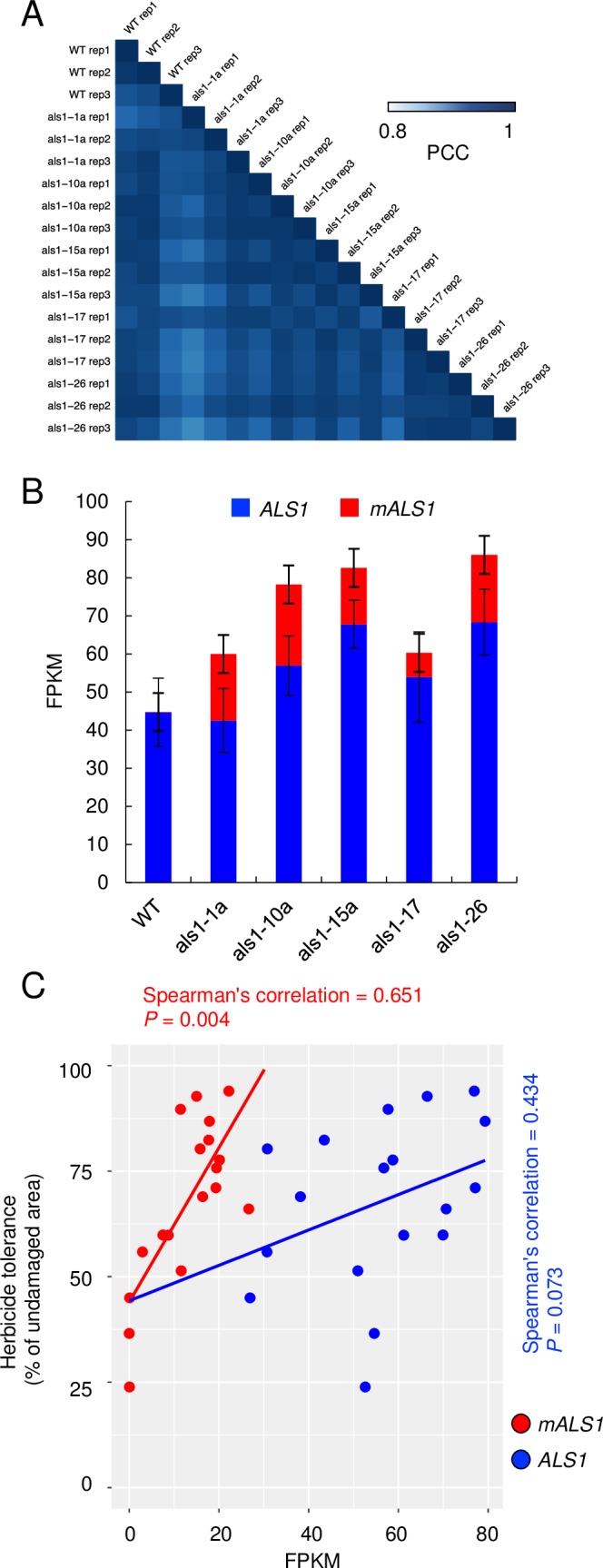
Transcriptome profiling in *mALS1* T-DNA transgenic lines. (A) Heatmap showing Pearson correlation coefficient (PCC) of gene expression among samples. (B) Allelic expression patterns of the *ALS1* and *mALS1* alleles. The mean allele specific expression at four single nucleotide polymorphism sites, A677G, C946T, G1688T and G1925C, was calculated in individual transgenic lines (mean ± SEM, n = 3). (C) Regression analysis between herbicide tolerance and allele specific *ALS1* expression. Spearman's correlation and *P*-values are labeled in red for the *mALS1* allele and blue for the WT allele.

To understand the impact of transformation on gene expression at the whole-genome level, we conducted RNA-seq analysis in young leaf tissue of WT and six transgenic lines selected for phenotypic variation (Fig 2 and S3 Table). Pairwise comparison of expression abundances revealed a high level of correlation not only among biological replicates (n = 3) in each transgenic line, but also across transgenic lines (Pearson correlation coefficient (PCC) ≥ 0.93 in any comparison; Fig 3A).

In the potato genome, there are two highly identical copies of *ALS* (*ALS1* and *ALS2)* and single nucleotide polymorphisms (SNPs) were used to quantitate the WT *ALS1* and *ALS2* and *mALS1* alleles (Figs 3B and S3). Due to the high sequence homology between ALS1 ALS2, expression of these two paralogs can not be differentiated and the expression level of the endogenous *ALS* genes shown here is likely from the combination of the two paralogs. As expected, expression of WT *ALS1* and *ALS2* alleles was higher than the hemizygous mutant *mALS1* allele which varied among individual transgenic lines, suggestive of a position effect of the T-DNA insertion in the genome and strength of expression. Expression of the *mALS1* allele was more correlated with herbicide tolerance (Spearman’s correlation = 0.651, and *P* = 0.004; Fig 3C) than the WT allele (Spearman’s correlation = 0.434, and *P* = 0.073). Collectively, these results indicate that expression of the transgene is correlated with herbicide tolerance of *mALS1* T-DNA transgenic lines.

We investigated effects of T-DNA insertion on expression of genes near the T-DNA insertion site using expression profiles. Consistent with the variability in the distance between the T-DNA and the closest gene (S2 Table), effects of the T-DNA insertion on expression of nearby genes varied (S2 Fig). The most striking example was observed in *als1-10a* in which the T-DNA was inserted 200 bp downstream of a gene encoding Small Auxin Up RNA (SAUR), *PGSC0003DMG400021980*, of which, expression increased by 1,400-fold in the transgenic line (S2 Fig). In juvenile leaf tissues in *A*. *thaliana*, multiple SAUR family genes were weakly expressed [46] suggesting that a potential repressor element of *PGSC0003DMG400021980* was impacted by T-DNA insertion in in *als1-10a*. Modest effects of the T-DNA insertion on expression of nearby genes were seen: *als1-1a*: *PGSC0003DMG400014340*; *als1-1a*: *PGSC0003DMG400014292*; *als1-10a*: *PGSC0003DMG400021979*; *als1-26*: *PGSC0003DMG402012908*; *als1-26*: *PGSC0003DMG402012907* (S2 Fig). These genes were relatively distant from T-DNA insertion sites, ranging from 2.8 kb to 15 kb. In addition, *als1-15a* in which the T-DNA was inserted 72.5 kb and 67.4 kb away from the flanking genes showed no effects on the expression of the flanking genes.

**Fig 4:**
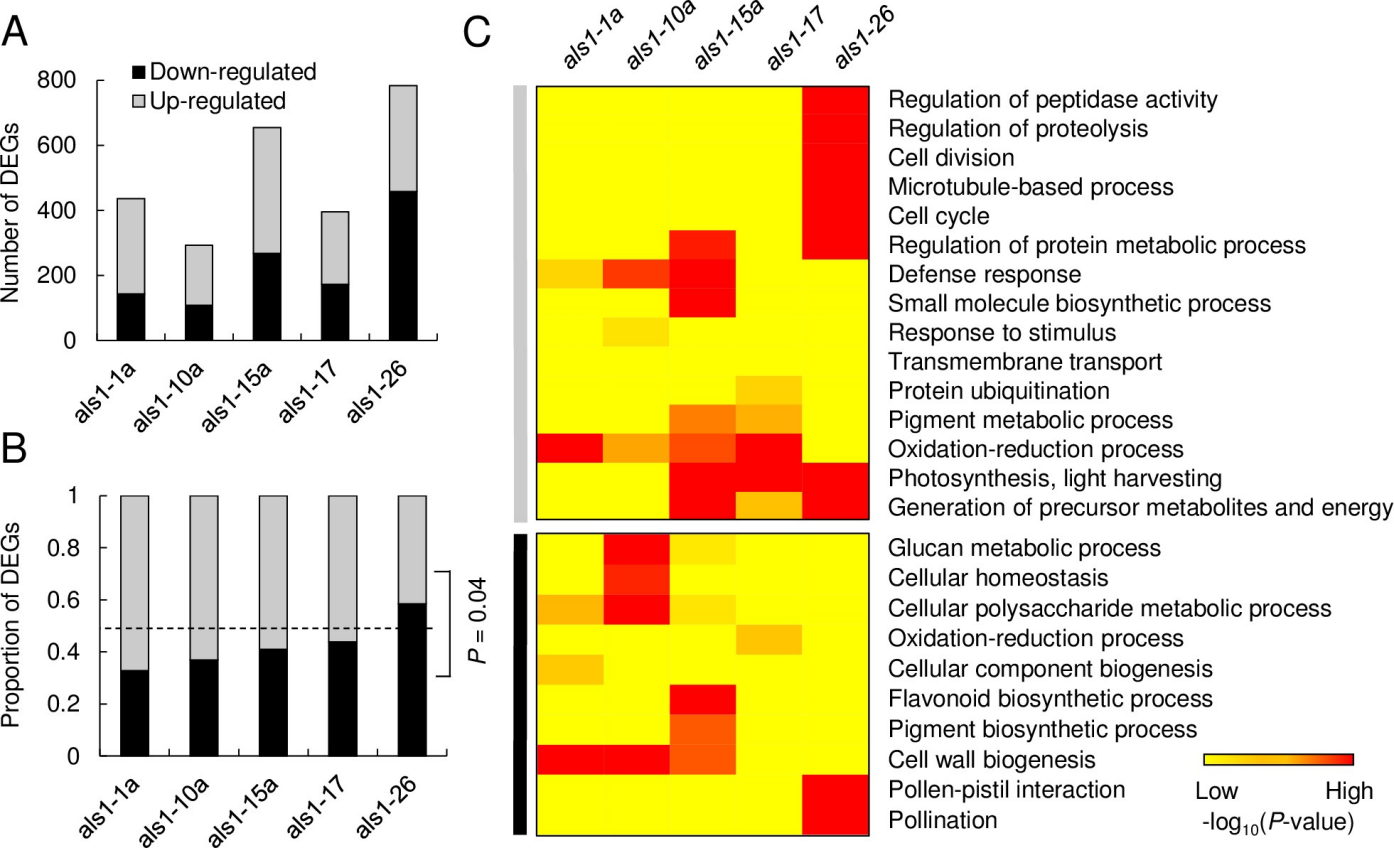
Differentially expressed genes (DEGs) in *mALS1* T-DNA transgenic lines relative to wild type. (A) Numbers of DEGs in individual transgenic lines. (B) Proportion of up-regulated and down-regulated DEGs in individual transgenic lines. Significant differences between the proportions of up-regulated DEGs and down-regulated DEGs was calculated using a two-tailed Student’s *t*-test. Dashed line indicates 50%. (C) Gene Ontology (GO) analysis of DEGs in individual *mALS1* T-DNA transgenic lines (adjusted *P*-value < 0.05, Hypergeometric test). A full list of GO terms significantly enriched is available in S5 Table. Down-regulated and up-regulated genes are indicated by grey and black bars on the left, respectively.

Differential expression analysis was performed by comparing gene expression data in each transgenic line to the WT using an adjusted *P* < 0.05 (S4 Table). Differentially expressed genes (DEGs) varied among the five transgenic lines analyzed (Fig 4A) with 783 DEGs in *als1-26*, of which, 325 were up-regulated and 458 were down-regulated. This contrasted with *als1-10a* with only 293 DEGs 185, of which, were up-regulated and 108 were down-regulated. Overall, all transgenic lines, with the exception of *als1-26*, showed a higher proportion of up-regulated DEGs compared to down-regulated DEGs (Fig 4B). The average proportion of DEGs up-regulated was significantly higher than DEGs down-regulated in all transgenic lines (*P* = 0.011), suggesting that the plant transformation had a higher impact on gene activation relative to gene repression.

Not surprisingly, the majority of DEGs were genotype-specific (S4 Fig) and GO analyses of up-regulated and down-regulated DEGs (Fig 4C) revealed that diverse biological pathways, including oxidation reduction process (GO:0055114), cellular homeostasis (GO:0019725), defense response (GO:0006952), pollen-pistil interaction (GO:0009875), pigment biosynthetic process (GO:0046148) and cell cycle (GO:0007049), were uniquely enriched in each transgenic line. Collectively, our transcriptome analysis demonstrated an extensive level of transcriptomic changes upon plant transformation, which were mainly unique to each transgenic line.

### Alteration in transcription factor activity among *mALS1* T-DNA transgenic lines

**Fig 5:**
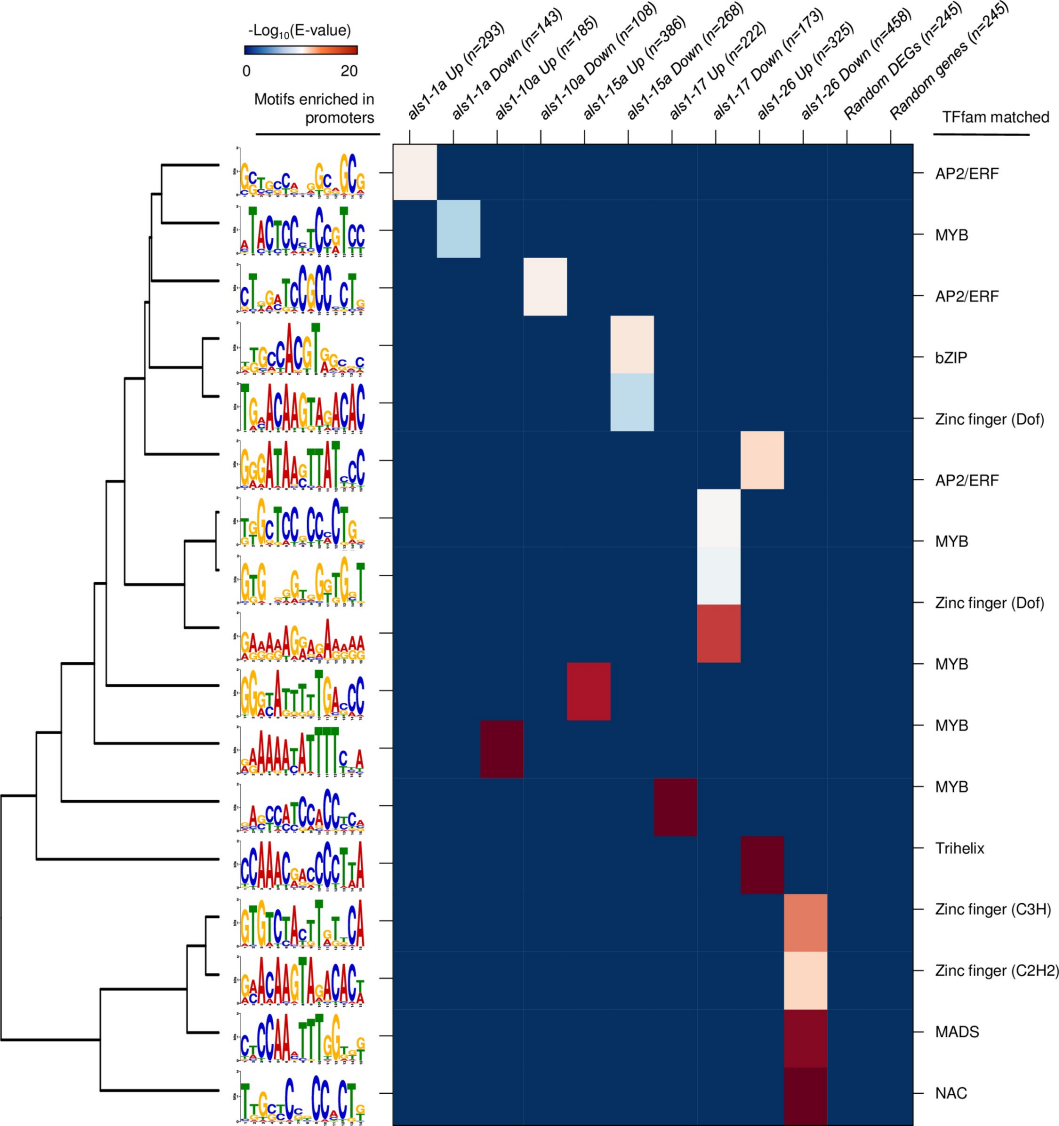
*De novo* motif analysis in core promoter regions of differentially expressed genes (DEGs). *De novo* motif analysis of promoters of DEGs using MEME [58]. Each genotype showed enrichment of unique transcription factor binding sites associated with various biological pathways. Motifs are indicated on the left of each row (See S6 Table). The best match to a transcription factor family for the motif in the cistrome database [37] is indicated to the right of each row. The median number of DEGs across the transgenic lines was 245 and we randomly selected 245 DEGs and 245 random annotated genes in the potato genome and performed motif analysis; no motifs were identified in either set. Full results for this figure are provided in S6 Table.

Transcriptional regulation by transcription factors (TFs), which could simultaneously regulate numerous downstream genes, is a primary level of gene regulation in most biological pathways [47]. Plant genomes are known to possess a higher expansion of TF families relative to other eukaryotes [48], which results in direct and indirect regulation of numerous biological pathways. We tested the possibility of TF regulation of DEGs by analyzing enrichment of potential *cis*-regulatory elements in their promoters. The core promoter sequences, defined here as 1000 bp upstream of transcription start site, of either up- or down-regulated DEGs were subjected to *de novo* motif analysis using MEME [36] (Fig 5A); similarities with the plant cistrome database [37] were further investigated using the TOMTOM tool [38]. Interestingly, at least one motif was significantly (E-value < 1.0E-07) and uniquely enriched in the promoters of DEGs in each transgenic line (S6 Table). Furthermore, all motifs, except the one found in up-regulated DEGs in *als1-26*, were statistically similar to binding elements of known TFs in plants, suggesting potential transcriptional regulation by TF activity to the elements in the *mASL1* T-DNA transgenic lines. For example, unique motifs statistically similar to binding elements of APETALA2/Ethylene Responsive Factor (AP2/ERF), which regulate stress responses and plant growth [49, 50], were enriched in the promoters of up-regulated DEGs in *als1-1a* but enriched in down-regulated DEGs in *als1-10a* and *als1-17*. Down-regulated DEGs in *als1-1a* and up-regulated DEGs in *als1-10a*, *als-15a*, *als1-17* and *als1-26* showed enrichments of motifs statistically similar with binding elements of MYB TF superfamily proteins, which regulate stress responses [51], circadian rhythms [52] and cell wall biogenesis [53]. In addition, unique motifs statistically similar with binding elements of bZIP, Trihelix, NAC and MADS box, DOF-, CCCH- and C2C2-zinc finger DNA-binding family proteins, which play roles in diverse biological pathways in plants [54–57], were enriched in down-regulated DEGs in *als1-15a* or up- or down-regulated DEGs in *als1-26*.

We then searched the plant transcription factor database (PlantTFDB 4.0) [59] to identify whether the TFs, whose binding sites were enriched in promoters of DEGs, were among the DEGs of each transgenic line. Indeed, 96 TFs from 17 TF families, including AP2, ERF, MYB and DOF, were included in DEGs of at least one transgenic line (S7 Table). Taken together, the presence of TFs within the DEGs and significant enrichment of their binding elements within the DEG sets strongly suggests that the gene-regulatory networks mediated by TFs were affected directly or indirectly by plant transformation, thereby contributing to transcriptomic and phenotypic variation. However, we do not exclude the possibility of other effects that could be raised during the clonal propagation (i.e., somaclonal variation). For example, DNA methylation levels could be altered on a genome scale affecting gene expression as shown in rice [18] and oil palm [60] processed through tissue culture without transformation. In addition, chromosomal rearrangements could occur, potentially leading to gene silencing through heterochromatin formation [16].

## Conclusion

Plant transformation in clonally propagated crops such as potato is a complex process, consisting of transgene delivery, random integration of T-DNA into the genome, selection, and regeneration from callus [6]. In this study, we investigated transcriptomic variation in a panel of *mALS1* transgenic lines derived from independent *Agrobacterium*-mediated transgenic events. Not surprisingly, the extent of herbicide tolerance was correlated with expression levels of the transgene, emphasizing the importance of the position effect and transcription levels of the transgene. We also consider other factors that may contribute to the transcriptomic variation such as epigenetic alterations caused by tissue culture and regeneration [16, 18], which were not explored in this study. Targeting T-DNA integration into well-characterized genomic regions with emerging genome editing technologies could address the impact of random T-DNA integration [9]. Not only was there an impact on gene expression in numerous biological pathways across the transgenic lines, a subset of these were associated with TF activities as diverse TF binding sites were enriched in core promoters of DEGs thereby adding an additional level of complexity to the transcriptomic impact.

## Supporting information

S1 Fig Southern blot of *NPTII Hind*III genomic DNA of thein genomes of *mALS1* T-DNA lines. Genomic DNA of *mALS1* T-DNA lines was digested with *Hind*III, transferred to a membrane, and hybridized with DIG-labeled *NPTII*. Primers used for the amplification of *NPTII* as a probe are provided in S2 Table.
(PPTX)

S2 Fig T-DNA insertion sites in selected transgenic lines. T-DNA insertion sites identified by inverse PCR in *als1-1a* (A), *als1-10a* (B), *als1-15a* (C) and *als1-26* (D). Fragments per kb exon model per million mapped reads (FPKM) of genes located on either side of T-DNA in the corresponding transgenic line and WT is shown.
(PPTX)

S3 Fig Allelic expression of *ALS1* and *mALS1* in transgenic lines. (A) Scheme of locations of four Single Nucleotide Polymorphisms (SNPs) introduced in the transgene, *mALS1*, of which, two were used for the analysis. (B) Allelic expression patterns of *ALS1* and *mALS1* at the two assayed sites. The frequency of each allele was measured at both sites, and an average was used to calculate the expression level in each transgenic line. Note, that no *ALS2* alleles were found at the two assayed sites.
(PPTX)

S4 Fig Differentially expressed genes (DEGs) in *mALS1* T-DNA transgenic lines relative to WT. Five-way Venn diagram showing unique and common DEGs in individual transgenic lines. Full lists of DEGs in individual transgenic lines are provided in S4 Table.
(PPTX)

S1 Table Locations of T-DNA insertion sites. (XLSX)

S2 Table List of primers used in transgenic screening, Southern blot analysis and inverse PCR. (XLSX)

S3 Table RNA-sequencing mapping summary. (XLSX)

S4 Table List of differentially expressed genes in each transgenic line. (XLSX)

S5 Table Enrichment of Gene Ontology (GO) groups of DEGs. Among ontology categories, biological process was used for the analysis.
(XLSX)

S6 Table *De novo* motif analysis summary. (XLSX)

S7 Table Presence of transcription factor (TF) genes within differentially expressed genes. The presence of TF genes is indicated by red boxes. The list of TF genes was obtained from PlantTFDB 4.0 [59].
(XLSX)
