# Supplementary material for: Transcriptome profiling of transgenic potato plants provides insights into variability caused by plant transformation
Source: PLoS One. 2018 Nov 8;13(11):e0206055. doi: 10.1371/journal.pone.0206055 (PMC6224046; doi:10.1371/journal.pone.0206055)
Supplement: S1 Fig — Genomic DNA of mALS1 T-DNA lines was digested with HindIII, transferred to a membrane, and hybridized with DIG-labeled NPTII. Primers used for the amplification of NPTII as a probe are provided in S2 Table. (PPTX) [file pone.0206055.s001.pptx]

## Slide 1
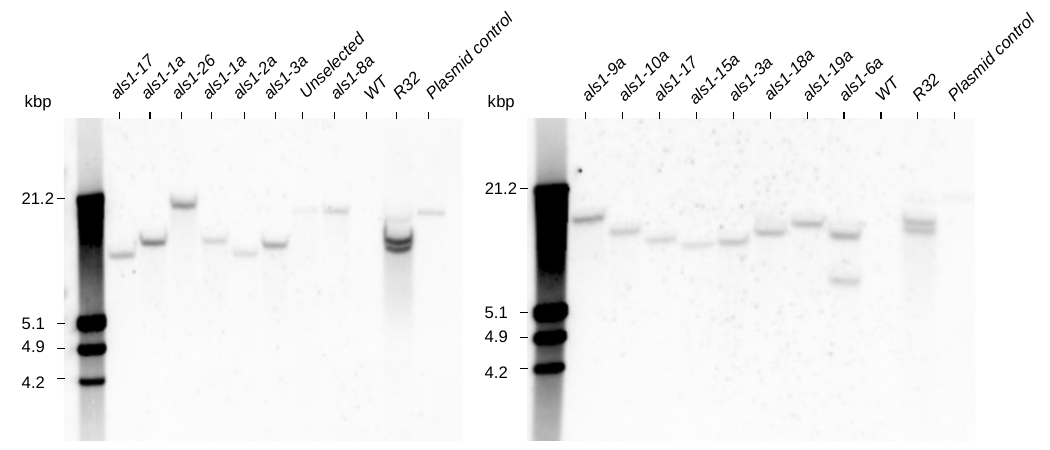

Plasmid control
Plasmid control
Unselected
als1-18a
als1-10a
als1-19a
als1-17
als1-1a
als1-26
als1-1a
als1-2a
als1-3a
als1-8a
als1-9a
als1-17
als1-15a
als1-3a
als1-6a
R32
R32
WT
WT
kbp
kbp
21.2
21.2
5.1
5.1
4.9
4.9
4.2
4.2
