# Supplementary material for: Transcriptome profiling of transgenic potato plants provides insights into variability caused by plant transformation
Source: PLoS One. 2018 Nov 8;13(11):e0206055. doi: 10.1371/journal.pone.0206055 (PMC6224046; doi:10.1371/journal.pone.0206055)
Supplement: S2 Fig — T-DNA insertion sites identified by inverse PCR in als1-1a (A), als1-10a (B), als1-15a (C) and als1-26 (D). Fragments per kb exon model per million mapped reads (FPKM) of genes located on either side of T-DNA in the corresponding transgenic line and WT is shown. (PPTX) [file pone.0206055.s002.pptx]

## Slide 1
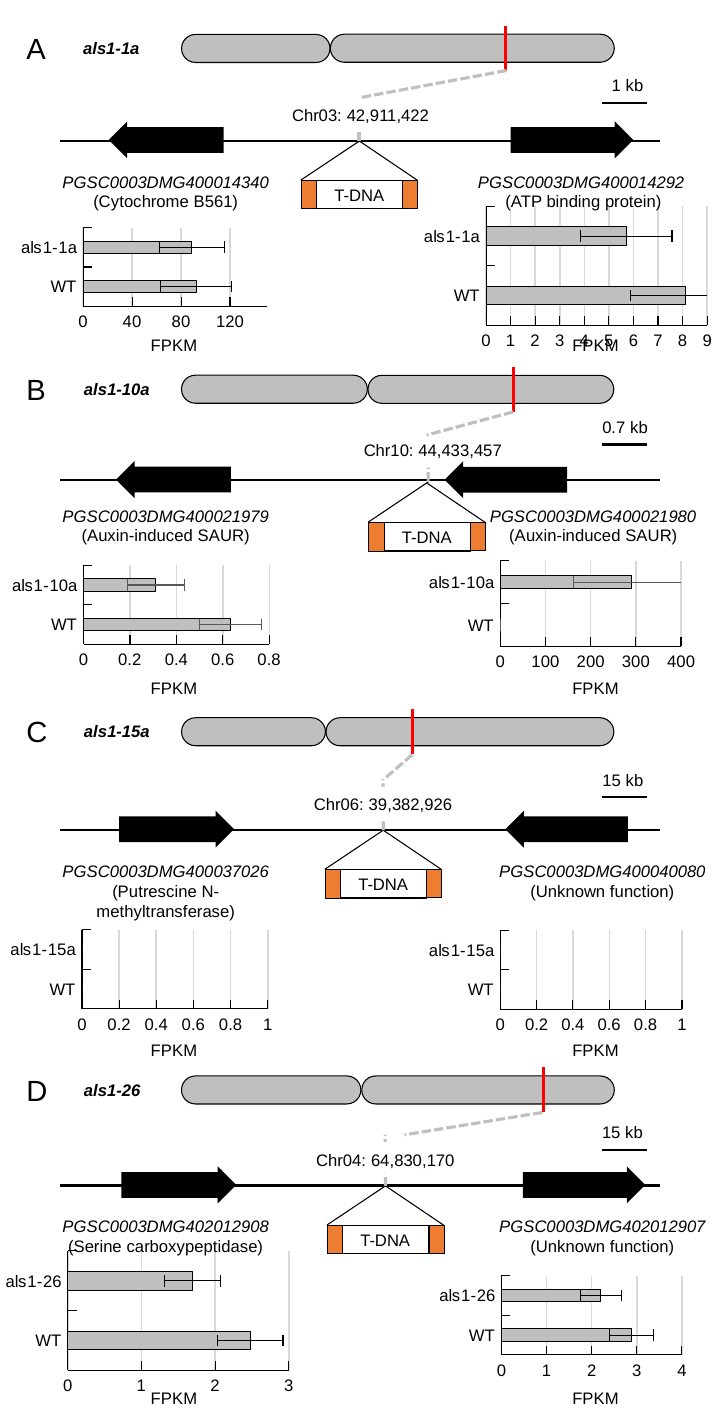

A
als1-1a
1 kb
Chr03: 42,911,422
PGSC0003DMG400014340
(Cytochrome B561)
PGSC0003DMG400014292
(ATP binding protein)
T-DNA
### Chart
| Category | avg |
|---|---|
| WT | 92.24876666666667 |
| als1-1a | 88.77896666666665 |
### Chart
| Category | avg |
|---|---|
| WT | 8.130056666666666 |
| als1-1a | 5.70026333333334 |FPKM
FPKM
B
als1-10a
0.7 kb
Chr10: 44,433,457
PGSC0003DMG400021979
(Auxin-induced SAUR)
PGSC0003DMG400021980
(Auxin-induced SAUR)
T-DNA
### Chart
| Category | avg |
|---|---|
| WT | 0.200334033333333 |
| als1-10a | 291.052 |
### Chart
| Category | avg |
|---|---|
| WT | 0.633679 |
| als1-10a | 0.3120153 |FPKM
FPKM
C
als1-15a
15 kb
Chr06: 39,382,926
PGSC0003DMG400040080
(Unknown function)
PGSC0003DMG400037026
(Putrescine N-methyltransferase)
T-DNA
### Chart
| Category | avg |
|---|---|
| WT | 0.0 |
| als1-15a | 0.0 |
### Chart
| Category | avg |
|---|---|
| WT | 0.0 |
| als1-15a | 0.0 |FPKM
FPKM
D
als1-26
15 kb
Chr04: 64,830,170
PGSC0003DMG402012908
(Serine carboxypeptidase)
PGSC0003DMG402012907
(Unknown function)
T-DNA
### Chart
| Category | avg |
|---|---|
| WT | 2.479506666666667 |
| als1-26 | 1.692396666666666 |
### Chart
| Category | avg |
|---|---|
| WT | 2.886776666666666 |
| als1-26 | 2.202066666666667 |FPKM
FPKM
