# Supplementary material for: Transcriptome profiling of transgenic potato plants provides insights into variability caused by plant transformation
Source: PLoS One. 2018 Nov 8;13(11):e0206055. doi: 10.1371/journal.pone.0206055 (PMC6224046; doi:10.1371/journal.pone.0206055)
Supplement: S3 Fig — (A) Scheme of locations of four Single Nucleotide Polymorphisms (SNPs) introduced in the transgene, mALS1, of which, two were used for the analysis. (B) Allelic expression patterns of ALS1 and mALS1 at the two assayed sites. The frequency of each allele was measured at both sites, and an average was used to calculate the expression level in each transgenic line. Note, that no ALS2 alleles were found at the two assayed sites. (PPTX) [file pone.0206055.s003.pptx]

## Slide 1
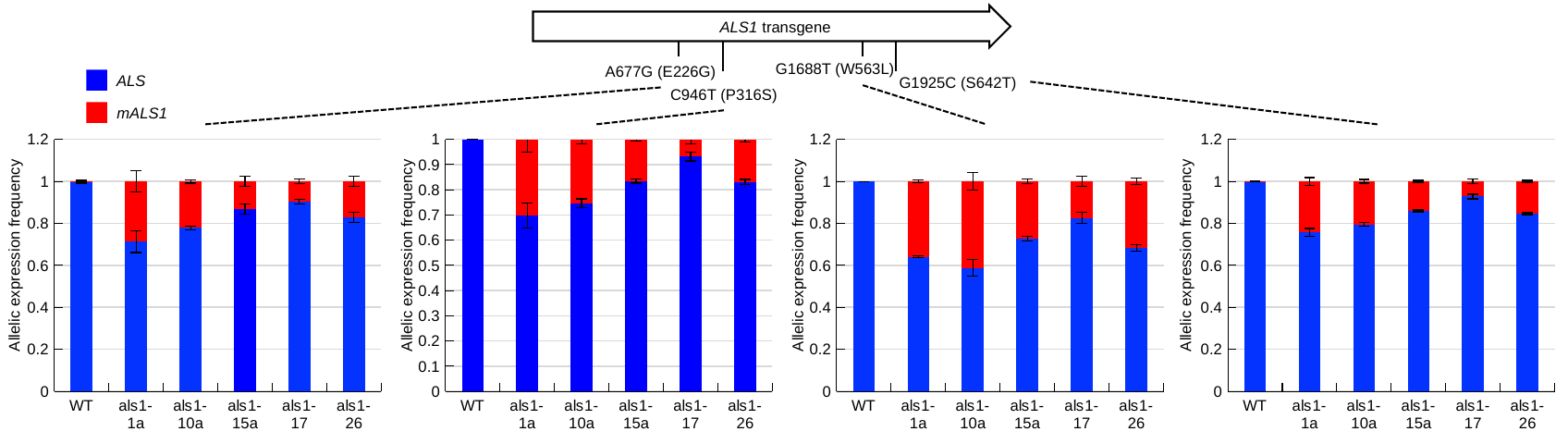

ALS1 transgene
G1688T (W563L)
A677G (E226G)
ALS
G1925C (S642T)
C946T (P316S)
mALS1
### Chart
| Category | | |
|---|---|---|
| WT | 0.9953703703703703 | 0.004629629629629629 |
| als1-1a | 0.7124114710321606 | 0.2875885289678393 |
| als1-10a | 0.7792403596428055 | 0.22075964035719456 |
| als1-15a | 0.8680286460575791 | 0.13197135394242085 |
| als1-17 | 0.9025260012025743 | 0.09747399879742578 |
| als1-26 | 0.8277209097642535 | 0.17227909023574658 |
### Chart
| Category | | |
|---|---|---|
| WT | 1.0 | 0.0 |
| als1-1a | 0.6979318567871453 | 0.30206814321285475 |
| als1-10a | 0.7468043095950073 | 0.25319569040499273 |
| als1-15a | 0.8342030707759608 | 0.1657969292240392 |
| als1-17 | 0.930806092537099 | 0.06919390746290094 |
| als1-26 | 0.8298539258053929 | 0.17014607419460706 |
### Chart
| Category | | |
|---|---|---|
| WT | 1.0 | 0.0 |
| als1-1a | 0.6407046795450068 | 0.3592953204549932 |
| als1-10a | 0.5871762035457916 | 0.4128237964542085 |
| als1-15a | 0.7261472039516389 | 0.27385279604836105 |
| als1-17 | 0.8259648427174199 | 0.17403515728257998 |
| als1-26 | 0.6832022941913052 | 0.3167977058086948 |
### Chart
| Category | | |
|---|---|---|
| WT | 0.9988465974625145 | 0.0011534025374855825 |
| als1-1a | 0.756148779086046 | 0.24385122091395392 |
| als1-10a | 0.7951197725169431 | 0.20488022748305693 |
| als1-15a | 0.8599725715468612 | 0.14002742845313873 |
| als1-17 | 0.9280745570553396 | 0.07192544294466052 |
| als1-26 | 0.8464850689431049 | 0.15351493105689518 |Allelic expression frequency
Allelic expression frequency
Allelic expression frequency
Allelic expression frequency
