# Supplementary figures and images for: Transcriptome profiling of transgenic potato plants provides insights into variability caused by plant transformation
Source: PLoS One. 2018 Nov 8;13(11):e0206055. doi: 10.1371/journal.pone.0206055 (PMC6224046; doi:10.1371/journal.pone.0206055)

## Slide 1
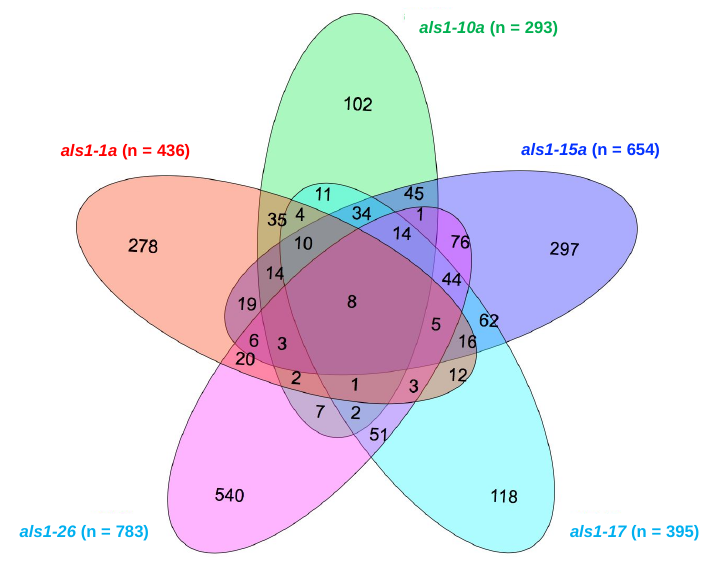

als1-10a (n = 293)
als1-15a (n = 654)
als1-1a (n = 436)
als1-26 (n = 783)
als1-17 (n = 395)

Supplement: S4 Fig — Five-way Venn diagram showing unique and common DEGs in individual transgenic lines. Full lists of DEGs in individual transgenic lines are provided in S4 Table. (PPTX) [file pone.0206055.s004.pptx]
